# On the dependence of the cardiac motion artifact on the breathing cycle in liver diffusion-weighted imaging

**DOI:** 10.1371/journal.pone.0239743

**Published:** 2020-10-01

**Authors:** Andreas Riexinger, Frederik Bernd Laun, Sebastian Bickelhaupt, Hannes Seuß, Michael Uder, Bernhard Hensel, Marc Saake

**Affiliations:** 1 Institute of Radiology, University Hospital Erlangen, Erlangen, Germany; 2 Center for Medical Physics and Engineering, Friedrich-Alexander-Universität Erlangen-Nürnberg (FAU), Erlangen, Germany; Medical University of Vienna, AUSTRIA

## Abstract

**Purpose:**

The purpose of this study was to investigate whether the cardiac motion artifact that regularly appears in diffusion-weighted imaging of the left liver lobe might be reduced by acquiring images in inspiration, when the coupling between heart and liver might be minimal.

**Materials and methods:**

43 patients with known or suspected focal liver lesions were examined at 1.5 T with breath hold acquisition, once in inspiration and once in expiration. Data were acquired with a diffusion-weighted echo planar imaging sequence and two b-values (b50 = 50 s/mm² and b800 = 800 s/mm²). The severity of the cardiac motion artifact in the left liver lobe was rated by two experienced radiologists for both b-values with a 5 point Likert scale. Additionally, the normalized signal S(b800)/S(b50) in the left liver lobe was computed. The Wilcoxon signed-rank test was used comparing the scores of the two readers obtained in inspiration and expiration, and to compare the normalized signal in inspiration and expiration.

**Results:**

The normalized signal in inspiration was slightly higher than in expiration (0.349±0.077 vs 0.336±0.058), which would indicate a slight reduction of the cardiac motion artifact, but this difference was not significant (p = 0.24). In the qualitative evaluation, the readers did not observe a significant difference for b50 (reader 1: p = 0.61; reader 2: p = 0.18). For b800, reader 1 observed a significant difference of small effect size favouring expiration (p = 0.03 with a difference of mean Likert scores of 0.27), while reader 2 observed no significant difference (p = 0.62).

**Conclusion:**

Acquiring the data in inspiration does not lead to a markedly reduced cardiac motion artifact in diffusion-weighted imaging of the left liver lobe and is in this regard not to be preferred over acquiring the data in expiration.

## Introduction

Diffusion weighted imaging (DWI) has been used widely for the detection of different pathologies of the liver, e.g. for the characterization of liver fibrosis and liver tumors, or the detection of liver metastases [1–5]. Compared to conventional MRI sequences such as, for example, T2-weighted sequences, DWI is of additional and high value, e.g. for the detection of lesions [6, 7]. Nonetheless, it is generally more prone to image artifacts.

In particular, motion artifacts represent a problem. They mostly arise from two sources, breathing and cardiac motion [8–10]. Breathing motion leads to the so-called stair-step artifacts [11], blurred images and a reduced sharpness, whereas the pulsation artifact originating from cardiac motion leads to a decreased or vanishing signal particularly in the left liver lobe [9]. To overcome the breathing motion problem, many studies aimed to find the best compensation technique, such as respiratory triggering (RT) or breath-hold (BH) imaging [1, 12–14]. Concerning the pulsation artifact, the left liver lobe was excluded from the analysis in several studies [8, 15]. However, the imaging of the entire liver is crucial for choosing the right treatment, e.g. if metastases are present [16]. Different approaches were investigated to reduce the pulsation artifact that partially build on advanced diffusion sequences like flow-compensated sequences [17, 18], which are not widely available and, more importantly, have drawbacks like a reduced b-value efficiency or a worsened black-blood contrast.

**Fig 1:**
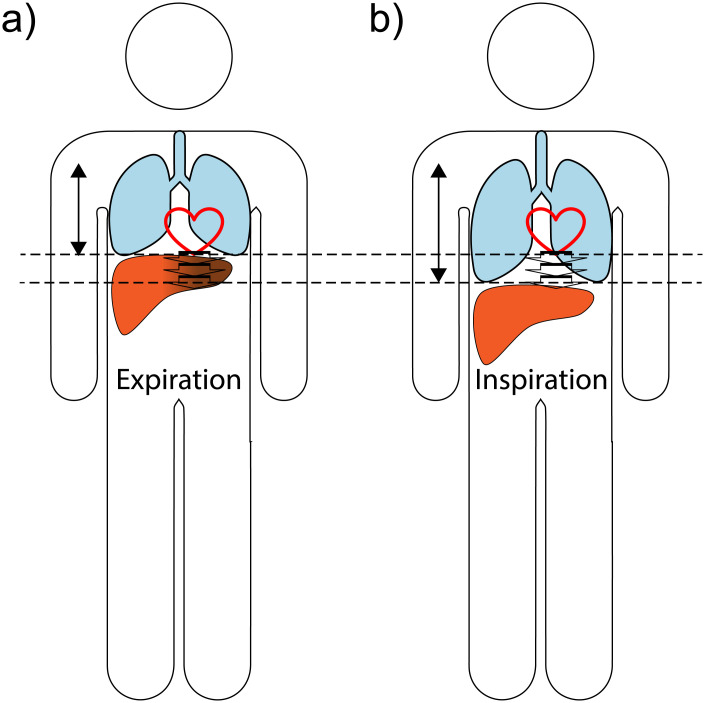
Visualization of the hypothesis underlying this study. a) Expiration: pulsation artifact present (signal void in the left liver lobe). b) Inspiration: reduced pulsation artifact.

A simple and easily applicable method to reduce the pulsation artifact in the left liver lobe would be of high value. This study is based on the hypothesis that the propagation of the pulsation artifact from the pulsating heart to the liver might depend on the breathing cycle because the relative position of heart and liver change while breathing. Although a significant inter-subject variability likely exists, the coupling might in general be smaller in the inspiration phase, which might thus be particularly suited for liver DWI (Fig 1). The aim of this study was to evaluate whether this hypothesis is correct.

## Material and methods

The study was approved by: Ethics committee of the Friedrich-Alexander-University Erlangen-Nürnberg Approval number: 276_19 B. Written informed consent was obtained.

### Study population

43 patients (23 male, 20 female, age 24–81 years, mean age 58.7 years) with known or suspected focal liver lesions were recruited for this study between October and December 2019. All participants gave written informed consent and the study was approved by the ethics committee of the Friedrich-Alexander-University Erlangen-Nürnberg.

### MR imaging protocol

All measurements were performed on a clinical 1.5 T scanner (MAGNETOM Aera, Siemens Healthcare, Erlangen, Germany) with an 18-channel anterior body coil in combination with a 32-channel spine coil. A single spin echo diffusion echo planar imaging (EPI) sequence provided by the vendor was used for the diffusion imaging. Images with the b-values b50 = 50 s/mm² and b800 = 800 s/mm² were acquired in inspired and expired breath hold, with two averages for the b800 images and one average for the b50 images. The diffusion mode was set to 3-scan-trace, with applied diffusion gradients along (1, 1, -0.5), (1, -0.5, 1), (-0.5, 1, 1) stated in the scanner coordinate system. 39 axial slices with a thickness of 5 mm, 1 mm gap, 2.4 x 2.4 mm² in-plane voxel size and field of view (FOV) 309 x 380 mm² were obtained with TE = 56 ms, TR = 1800 ms, parallel imaging (GRAPPA) factor 2, and SPAIR fat suppression. The vendor-provided surface coil intensity correction option was used to compensate for surface coil flare. Moreover, the vendor-provided dynamic field correction method was used to compensate for eddy current artifacts. The acquisition time was 10:08 min for both breath hold schemes in total.

### Visual assessment of the datasets

First, the image quality was checked and images with a non-diagnostic quality were excluded from the further analysis. The reason for excluding the images was recorded. The two-tailed Barnard’s exact test was performed to test for a relation between exclusion and breath-hold technique.

The severity of the cardiac motion artifact was evaluated with a Likert scale ranging from 1 (poor) to 5 (excellent) by visual assessment (c.f. Table 1). The trace-weighted images were evaluated by two radiologists with 6 and 8 years of experience in abdominal MRI (S. B. and H. S., respectively). For the inspiration data and for the expiration data, respectively, one rating was given for all slices by each reviewer. For data analysis, both ratings were pooled.

**Table 1:** Definition of the Likert score for the visual assessment of the cardiac motion artifact.

(a)	Score	Cardiac motion artifact
	1	Left liver lobe not identifiable
	2	More than one large black hole visible
	3	Generally strong signal loss or one large black hole or frequent small black holes.
	4	Generally weak signal loss and no large black hole and if small black holes are present, they are not frequent.
	5	No signal loss visible

### Quantitative analysis of the datasets

To assess the severity of the cardiac motion artifact quantitatively, polygon shaped regions of interest (ROIs) were drawn by A.R. on each b50 image (inspiration, expiration) in each slice covering as much of the left liver lobe as possible in the respective slice. These ROIs were then copied to the respective b800 image. Slices without liver parenchyma were neglected. The average signal was calculated for each patient, b-value, and breathing scheme by taking the mean pixel value of the whole volume. The average values from the b800 images was then normalized with the average signal of the corresponding b50 images. These computations were performed in MITK v2016.3 (German Cancer Research Center, Heidelberg, Germany) and Excel 2016 (Microsoft, Redmond, United States). The Spearman’s rank correlation coefficient was calculated to identify the correlation between inspiration and expiration ratings.

### Statistics

The Likert scores of inspiration and expiration data were compared using the Wilcoxon signed-rank test for each b-value and reader. For the quantitative analysis, the signals at b = 800 s/mm² for inspiration and expiration data were compared using the Wilcoxon signed-rank test. In both tests, p < 0.05 was considered to be significant. Cohen’s kappa coefficient κ was used to assess the interreader agreement. κ was interpreted as follows: 0 <κ≤ 0.2 = slight agreement, 0.2 <κ≤ 0.4 = fair agreement, 0.4 <κ≤ 0.6 = moderate agreement, 0.6 <κ≤ 0.8 = substantial agreement, 0.8 <κ≤ 1.0 = almost perfect agreement, and κ = 1 as perfect agreement.

## Results

### Image quality check

Seven patients were excluded from the further evaluation due to severe breathing motion artifacts in the inspiration breath-hold images (6 patients) or due to an extremely low signal in the whole DWI data, potentially related to incorrect measurement adjustments (1 patient). According to the Barnards exact test, the exclusion of patients is related to the breath-hold scheme (p = 0.0479). In some images, residual fat artifacts were present, which had no effect on the clinical evaluation. Thus 36 patients were further evaluated within this study.

### Representative images

**Fig 2:**
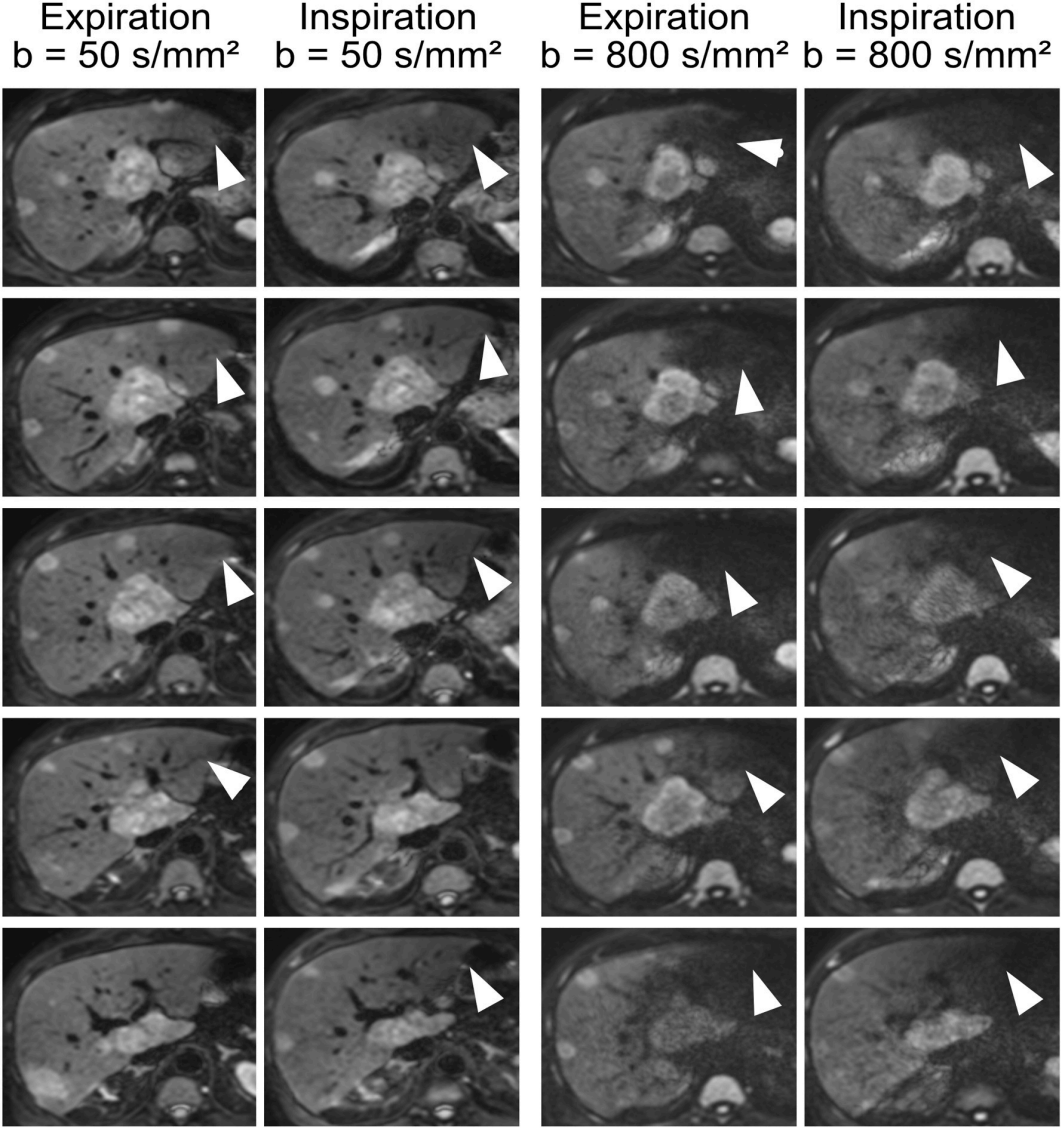
Diffusion weighted images of one patient acquired with breath-hold in expiration and inspiration at two b-values. Each row shows the same slice. The pulsation artifact is marked by arrows.

Fig 2 shows representative trace-weighted diffusion weighted images of one patient acquired with breath-hold in expiration and in inspiration at b = 50 and 800 s/mm². Each row shows the same slice. The pulsation artifact in the left liver lobe is present in all images at b = 800 s/mm² and sometimes at b = 50 s/mm² (arrows). At the lower b-value, regions of reduced signal can be identified. At b = 800 s/mm², the pulsation artifact is markedly stronger and the left liver lobe is often hardly identifiable. Both, in inspiration and expiration, the pulsation artifact is equally present.

### Qualitative analysis

κ was <0.2 between both reader for the b50 and b800 images. One explanation is that reader 2 (H.S.) viewed the data more benevolently and generally assigned higher scores. For example, for the pulsation artifact at b800, reader 1 scored 2.05 (inspiration) and 2.32 (expiration) on average, while reader 2 scored 3.50 (inspiration) and 3.45 (expiration).

**Fig 3:**
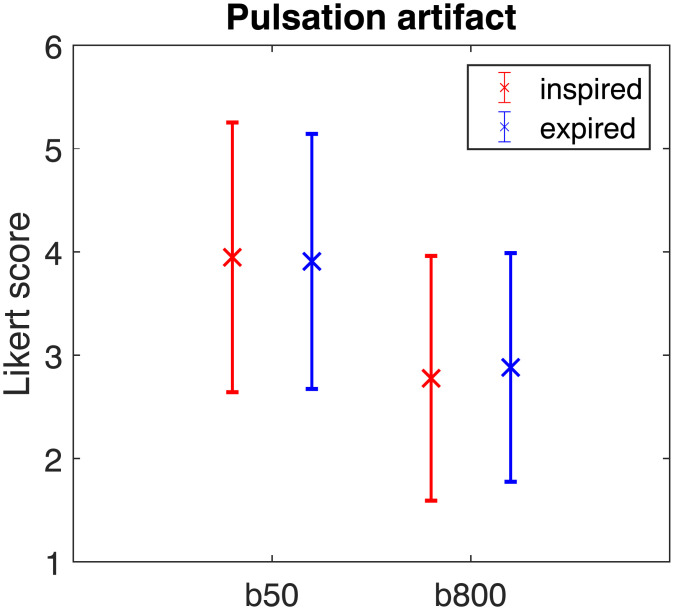
Mean Likert scores and standard deviation of the pulsation artifact obtained for inspiration (red) and expiration (blue). The scores of the two readers were pooled.

**Fig 4:**
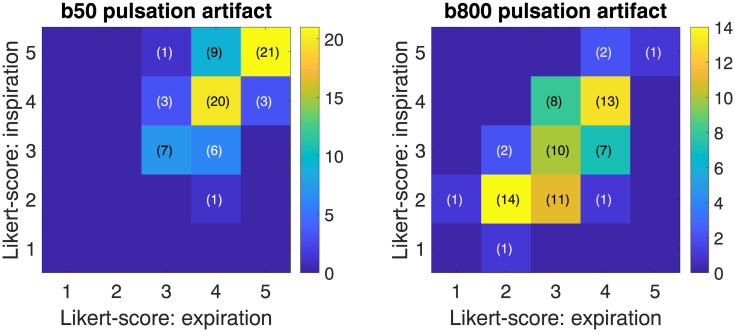
Plots comparing Likert scores for the pulsation artifacts in inspiration and expiration. X-axis: expiration Likert score; Y-axis: inspiration Likert score. The scores of both readers were pooled in this plot.

Mean Likert scores for the pulsation artifact are shown in Fig 3 pooling the scores of both readers. Fig 4 additionally visualizes the correlation between the Likert scores for inspiration and expiration data. Although the data is nominal, the quantitative operation of computing the mean was used in Fig 3 for simplicity. At b800, the mean Likert score for the pulsation artifact is markedly reduced compared to b50, indicating stronger artifacts. However, the mean reader-averaged Likert score is almost identical for inspiration and expiration data at b50 and b800 (b50: inspired 3.95, expired 3.91; b800: inspired 2.78, expired 2.88).

As mentioned above, both readers agreed in that no significant differences existed for the b50 images (reader 1: p = 0.61; reader 2: p = 0.18). For b800, reader 2 observed no significant difference (p = 0.62), unlike reader 1, who reported a significant difference of small effect size favouring expiration (p = 0.03 with a difference of mean Likert scores of 0.27).

### Quantitative analysis

**Fig 5:**
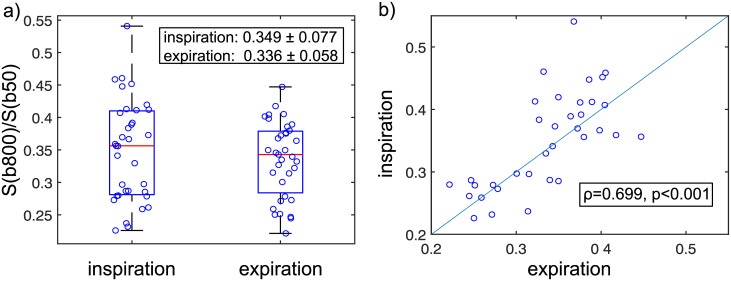
Normalized signal intensities measured in the left liver lobe in inspiration and expiration. S(b800) is the signal measured at b = 800 s/mm² and S(b50) is the signal measured at b = 50 s/mm². Each marker represents one patient. a) Boxplots. b) Scatter-plot of the same data shown in (a). The Spearman rank correlation coefficient is stated.

Fig 5 shows the normalized signal measured in the left liver lobe. The normalized signal in inspiration was slightly higher than in expiration (0.349±0.077 vs 0.336±0.058). This difference was not significant (p = 0.24). This indicates that the pulsation artifact in inspiration is not reduced, or, at best, only slightly reduced.

## Discussion

This study investigated whether diffusion-weighted imaging of the liver in inspiration leads to a reduction of the cardiac motion artifact in the left liver lobe. The quantitative evaluation slightly favors acquiring images in inspiration, whereas the qualitative evaluation showed no significant differences. The supposed significant increase in image quality was not observed for the inspiration DWI data. It thus seems that little to no difference between DWI images acquired in inspiration and expiration exists and that its influence on potential cardiac motion artifacts might not be a relevant variable for the decision whether to acquire DWI of the liver in inspiration or expiration.

Studies investigating further approaches to increase the image quality of the left liver lobe in DWI are still warranted. Achieving such an increase in image quality could improve the diagnostic performance and reliability of the method. At the moment, based upon the limitation of DWI in the left liver lobe and also in the liver dome, there is an added value of contrast-enhanced dynamic imaging, and, in particular, for gadoxetic acid-enhanced MRI in combination with DWI, especially in the preoperative evaluation and detection of liver metastases [19].

We acquired the data with breath-hold (BH) acquisition, and not with navigator-triggered (NT) techniques, because the used sequence did not allow this in the case of inspiration. Generally, BH as an alternative to NT and respirator triggering (RT, i.e. via an external trigger) seems to be less recommendable, in particular for the detection of focal liver lesions (FLL). Kandpal et al. found that RT should be preferred over BH acquisition for the assessment of FLL because it provides a better image quality and higher lesion to liver CNR [14]. The decreased liver to lesion contrast using BH acquisition was also reported by Taouli et al. [20] and by Choi et al. [1].

Most NT approaches aim at acquiring the data in expiration (e.g. [1, 12, 21, 22]). Our data does not suggest that moving away from this practice is advisable. Not only did the pulsation artifact not improve in inspiration, we moreover found six inspiration datasets that did not pass the initial quality check. This may be related to difficulties of some patients to properly hold their breath in inspiration in a reproducible fashion in consecutive breath-hold cycles and might be different for NT. Larsen et al. similarly reported that BH acquisition in inspiration did not pass their quality check in 5 out of 11 cases [23]. Little evidence appears to exist in the literature that this finding would be different for NT or RT.

Addressing the cardiac motion artifact, other approaches were tested in the past. E.g. using electrocardiogram (ECG) triggering is an option [24], which can, however, be problematic in DWI because fast switching gradient fields in DWI may degrade the quality of the ECG. Post-processing methods such as using a weighted averaging over different diffusion directions [25] or the stronger weighting of large signals measured in multiple acquisitions [26] are promising approaches, as long as a sufficient number of images does not suffer from an almost complete signal loss. A further promising approach is the use of partially or fully flow-compensated diffusion encodings [10, 17, 18]. Those, however, may suffer from bright blood signal at low b-values [17, 27, 28] and of prolonged echo times because the flow-compensated encoding decreases the b-value efficiency.

We acknowledge some limitations. First, we only rated the image quality of the left liver lobe and neglected the right liver lobe because the cardiac motion artifact is mostly present in the left liver lobe. Second, it is difficult to draw a ROI including the entire left liver lobe when the signal is low due to the pulsation artifact, which may have led to a limited precision of the quantitative evaluation. Third, the interreader agreement was only slight. The low κ values might have been improved by introducing a training session with both readers, which we had not performed. Fourth, we used only a single scanner of one vendor at one site. Potentially, additional data acquired at different fields and different scanners might have revealed significant differences. It seems, however, unlikely that such difference could be strong enough to be relevant in clinical practice. Fifth, we evaluated only breath hold data. Potentially, the situation might be different when using gated acquisitions. Lastly, we did not perform further investigations on why the acquiring the data in inspiration did not result in a reduced cardiac motion artifact. One approach to further elucidate this somewhat unanticipated result might be to acquire time-resolved data of the motion fields in the liver and to assess the dephasing one would expect given a certain diffusion encoding. We considered this task to be beyond the scope of this work.

In conclusion, acquiring the data in inspiration does not lead to a markedly reduced cardiac motion artifact and is in this regard not to be preferred over acquiring the data in expiration.

## References

[1] Choi JS, Kim MJ, Chung YE, Kim KA, Choi JY, Lim JS, et al Comparison of breathhold, navigator-triggered, and free-breathing diffusion-weighted MRI for focal hepatic lesions. Journal of Magnetic Resonance Imaging. 2013;38(1):109–18. 10.1002/jmri.23949 23188562

[2] Koh DM, Collins DJ. Diffusion-weighted MRI in the body: applications and challenges in oncology. AJR Am J Roentgenol. 2007;188(6):1622–35. 10.2214/AJR.06.1403 17515386

[3] Qayyum A. Diffusion-weighted imaging in the abdomen and pelvis: concepts and applications. Radiographics. 2009;29(6):1797–810. 10.1148/rg.296095521 19959522 PMC6939846

[4] Taouli B, Chouli M, Martin AJ, Qayyum A, Coakley FV, Vilgrain V. Chronic hepatitis: role of diffusion-weighted imaging and diffusion tensor imaging for the diagnosis of liver fibrosis and inflammation. J Magn Reson Imaging. 2008;28(1):89–95. 10.1002/jmri.21227 18581382

[5] Taouli B, Koh DM. Diffusion-weighted MR imaging of the liver. Radiology. 2010;254(1):47–66. 10.1148/radiol.09090021 20032142

[6] Bruegel M, Gaa J, Waldt S, Woertler K, Holzapfel K, Kiefer B, et al Diagnosis of Hepatic Metastasis: Comparison of Respiration-Triggered Diffusion-Weighted Echo-Planar MRI and Five T2-Weighted Turbo Spin-Echo Sequences. Am J Roentgenol. 2008;191(5):1421–9. 10.2214/AJR.07.3279 18941080

[7] Parikh T, Drew SJ, Lee VS, Wong S, Hecht EM, Babb JS, et al Focal liver lesion detection and characterization with diffusion-weighted MR imaging: comparison with standard breath-hold T2-weighted imaging. Radiology. 2008;246(3):812–22. 10.1148/radiol.2463070432 18223123

[8] Kwee TC, Takahara T, Koh DM, Nievelstein RA, Luijten PR. Comparison and reproducibility of ADC measurements in breathhold, respiratory triggered, and free-breathing diffusion-weighted MR imaging of the liver. J Magn Reson Imaging. 2008;28(5):1141–8. 10.1002/jmri.21569 18972355

[9] Kwee TC, Takahara T, Niwa T, Ivancevic MK, Herigault G, Van Cauteren M, et al Influence of cardiac motion on diffusion-weighted magnetic resonance imaging of the liver. MAGMA. 2009;22(5):319–25. 10.1007/s10334-009-0183-1 19727877

[10] Ozaki M, Inoue Y, Miyati T, Hata H, Mizukami S, Komi S, et al Motion artifact reduction of diffusion-weighted MRI of the liver: use of velocity-compensated diffusion gradients combined with tetrahedral gradients. J Magn Reson Imaging. 2013;37(1):172–8. 10.1002/jmri.23796 22987784

[11] Ivancevic MK, Kwee TC, Takahara T, Ogino T, Hussain HK, Liu PS, et al Diffusion-Weighted MR Imaging of the Liver at 3.0 Tesla Using Tracking Only Navigator Echo (TRON): a Feasibility Study. Journal of Magnetic Resonance Imaging. 2009;30(5):1027–33. 10.1002/jmri.21939 19856431

[12] Bouchaibi SE, Coenegrachts K, Bali MA, Absil J, Metens T, Matos C. Focal liver lesions detection: Comparison of respiratory-triggering, triggering and tracking navigator and tracking-only navigator in diffusion-weighted imaging. Eur J Radiol. 2015;84(10):1857–65. 10.1016/j.ejrad.2015.06.018 26119802

[13] Chen X, Qin L, Pan D, Huang Y, Yan L, Wang G, et al Liver diffusion-weighted MR imaging: reproducibility comparison of ADC measurements obtained with multiple breath-hold, free-breathing, respiratory-triggered, and navigator-triggered techniques. Radiology. 2014;271(1):113–25. 10.1148/radiol.13131572 24475860

[14] Kandpal H, Sharma R, Madhusudhan KS, Kapoor KS. Respiratory-triggered versus breath-hold diffusion-weighted MRI of liver lesions: comparison of image quality and apparent diffusion coefficient values. AJR Am J Roentgenol. 2009;192(4):915–22. 10.2214/AJR.08.1260 19304695

[15] Dyvorne HA, Galea N, Nevers T, Fiel MI, Carpenter D, Wong E, et al Diffusion-weighted imaging of the liver with multiple b values: effect of diffusion gradient polarity and breathing acquisition on image quality and intravoxel incoherent motion parameters—a pilot study. Radiology. 2013;266(3):920–9. 10.1148/radiol.12120686 23220895 PMC3579172

[16] Yacoub JH, Elsayes KM, Fowler KJ, Hecht EM, Mitchell DG, Santillan C, et al Pitfalls in liver MRI: Technical approach to avoiding misdiagnosis and improving image quality. J Magn Reson Imaging. 2019;49(1):41–58. 10.1002/jmri.26343 30295343

[17] Rauh SS, Riexinger AJ, Ohlmeyer S, Hammon M, Saake M, Stemmer A, et al A mixed waveform protocol for reduction of the cardiac motion artifact in black-blood diffusion-weighted imaging of the liver. Magn Reson Imaging. 2020;67:59–68. 10.1016/j.mri.2019.12.011 31923466

[18] Zhang Y, Pena-Nogales O, Holmes JH, Hernando D. Motion-robust and blood-suppressed M1-optimized diffusion MR imaging of the liver. Magn Reson Med. 2019;82(1):302–11. 10.1002/mrm.27735 30859628

[19] Vilgrain V, Esvan M, Ronot M, Caumont-Prim A, Aube C, Chatellier G. A meta-analysis of diffusion-weighted and gadoxetic acid-enhanced MR imaging for the detection of liver metastases. Eur Radiol. 2016;26(12):4595–615. 10.1007/s00330-016-4250-5 26883327

[20] Taouli B, Sandberg A, Stemmer A, Parikh T, Wong S, Xu J, et al Diffusion-Weighted Imaging of the Liver: Comparison of Navigator Triggered and Breathhold Acquisitions. Journal of Magnetic Resonance Imaging. 2009;30(3):561–8. 10.1002/jmri.21876 19711402

[21] Jerome NP, Orton MR, d’Arcy JA, Collins DJ, Koh DM, Leach MO. Comparison of Free-Breathing With Navigator-Controlled Acquisition Regimes in Abdominal Diffusion-Weighted Magnetic Resonance Images: Effect on ADC and IVIM Statistics. Journal of Magnetic Resonance Imaging. 2014;39(1):235–40. 10.1002/jmri.24140 23580454

[22] Lee Y, Lee SS, Kim N, Kim E, Kim YJ, Yun SC, et al Intravoxel incoherent motion diffusion-weighted MR imaging of the liver: effect of triggering methods on regional variability and measurement repeatability of quantitative parameters. Radiology. 2015;274(2):405–15. 10.1148/radiol.14140759 25232802

[23] Larsen NE, Haack S, Larsen LPS, Pedersen EM. Quantitative liver ADC measurements using diffusion-weighted MRI at 3 Tesla: evaluation of reproducibility and perfusion dependence using different techniques for respiratory compensation. Magn Reson Mater Phy. 2013;26(5):431–42. 10.1007/s10334-013-0375-6 23483359

[24] Xiang Z, Ai Z, Liang J, Li G, Zhu X, Yan X. Evaluation of Regional Variability and Measurement Reproducibility of Intravoxel Incoherent Motion Diffusion Weighted Imaging Using a Cardiac Stationary Phase Based ECG Trigger Method. Biomed Res Int. 2018;2018:4604218 10.1155/2018/4604218 29850518 PMC5932501

[25] Ichikawa S, Motosugi U, Tamada D, Wakayama T, Sato K, Funayama S, et al Improving the Quality of Diffusion-weighted Imaging of the Left Hepatic Lobe Using Weighted Averaging of Signals from Multiple Excitations. Magn Reson Med Sci. 2019;18(3):225–32. 10.2463/mrms.mp.2018-0085 30555108 PMC6630049

[26] Liau J, Lee J, Schroeder ME, Sirlin CB, Bydder M. Cardiac motion in diffusion-weighted MRI of the liver: artifact and a method of correction. J Magn Reson Imaging. 2012;35(2):318–27. 10.1002/jmri.22816 21959926 PMC3252483

[27] Gurney-Champion OJ, Rauh SS, Harrington K, Oelfke U, Laun FB, Wetscherek A. Optimal acquisition scheme for flow-compensated intravoxel incoherent motion diffusion-weighted imaging in the abdomen: An accurate and precise clinically feasible protocol. Magn Reson Med. 2020;83(3):1003–15. 10.1002/mrm.27990 31566262 PMC6899942

[28] Wetscherek A, Stieltjes B, Laun FB. Flow-compensated intravoxel incoherent motion diffusion imaging. Magn Reson Med. 2015;74(2):410–9. 10.1002/mrm.25410 25116325

